# CARE Registry retention: Insights from focus groups with Asian American, Native Hawaiian, and Pacific Islander populations in the context of Alzheimer's disease and related dementias research

**DOI:** 10.1002/alz70860_105110

**Published:** 2025-12-23

**Authors:** Van Ta Park, Janice Y. Tsoh, Bora Nam, Marian Tzuang, Nicole Phan, Chandra Chak, Nimita Gaggar, Kenny Li, Oanh L. Meyer, Arnab Mukherjea, Christy Nishita, Poki'i Balaz, Joyce R. Javier, Hye‐Won Shin, Josh D Grill, Cati Brown Johnson

**Affiliations:** ^1^ Asian American Research Center on Health (ARCH), University of California San Francisco, San Francisco, CA, USA; ^2^ University of California San Francisco School of Nursing, San Francisco, CA, USA; ^3^ University of California San Francisco School of Medicine, San Francisco, CA, USA; ^4^ The UC Irvine Institute for Memory Impairments and Neurological Disorders, Irvine, CA, USA; ^5^ University of California, Davis School of Medicine, Sacramento, CA, USA; ^6^ California State University, East Bay, Hayward, CA, USA; ^7^ Center on Aging, University of Hawaii, Honolulu, HI, USA; ^8^ Kokua Kalihi Valley Comprehensive Family Services, Honolulu, HI, USA; ^9^ Department of Health Systems Science, Kaiser Permanente Bernard J. Tyson School of Medicine, Pasadena, CA, USA; ^10^ Somang Society, Cypress, CA, USA; ^11^ Evaluation Sciences Unit, Division of Primary Care and Population Health, Stanford School of Medicine, Stanford, CA, USA

## Abstract

**Background:**

The Collaborative Approach for AANHPI Research and Education (CARE) registry aims to address underrepresentation of Asian American, Native Hawaiian, and Pacific Islander (AANHPI) populations in aging, Alzheimer's disease and related dementias, and caregiving research (*n* = 10,485 AANHPI adults). To inform retention efforts, we conducted focus groups with participants who have been enrolled in CARE for at least 1 year.

**Method:**

We conducted 13 groups (*N* = 71) in English via Zoom during Fall 2024. Trained culturally congruent CARE partners/staff conducted the semi‐structured focus groups. Topics included: understanding and experience with CARE; registry retention; communication framing; overall recommendations. We conducted rapid qualitative analyses following the Stanford Lightning Report methods: Plus (what's working), Delta (what needs to change), and Insight (participant insights/ideas) to abstract findings.

**Result:**

Focus groups included 12 Chinese, 11 Asian Indian, 18 Filipino, 8 Japanese, 8 Korean, 6 Native Hawaiian, and 8 Vietnamese participants. 62% were women; 28.2% were ≥65 years old; 46% were family caregivers.

Plus: Participants across ethnicities expressed excitement to contribute to AANHPI representation, and some groups connected this to giving back as a major motivator for retention: “if I can help in any way… assisting members of our community…” (Asian Indian participant).

Delta: To facilitation continuation, participants wanted to contribute by gaining deeper understanding of their impact and more opportunities for research involvement: “[I] want to hear about how I’m making a difference through CARE” (Asian Indian participant). Regarding risks for discontinuation, participants identified future potential data privacy concerns: “if CARE was privatized or sold to a corporation, then I would probably be disincentivized to further participate” (Korean participant).

Insights/ideas: To improve communication efficiencies, while communication modality preferences (e.g. texting vs. email) varied, many suggested promoting testimonials from CARE participants (via social media/YouTube) as a retention method.

**Conclusion:**

Rapid qualitative analysis of focus groups provided actionable insights to inform and improve CARE retention strategies. Focus groups in AANHPI languages are planned for Spring 2025. Future communication regarding retention can emphasize these focus group themes for CARE: the importance of AANHPI representation in research and science and giving back to the community.

